# Divergent role of PIDA and PIFA in the AlX_3_ (X = Cl, Br) halogenation of 2-naphthol: a mechanistic study

**DOI:** 10.3762/bjoc.20.141

**Published:** 2024-07-15

**Authors:** Kevin A Juárez-Ornelas, Manuel Solís-Hernández, Pedro Navarro-Santos, J Oscar C Jiménez-Halla, César R Solorio-Alvarado

**Affiliations:** 1 Departamento de Química, División de Ciencias Naturales y Exactas, Universidad de Guanajuato, Campus Gto, Noria Alta S/N 36050, Guanajuato, México; 2 CONAHCYT - Instituto de Investigaciones Químico Biológicas, Universidad Michoacana de San Nicolás de Hidalgo, Avenida Francisco J. Múgica S/N 58030, Morelia, Michoacán, México

**Keywords:** aromatic bromination, aromatic chlorination, density functional theory (DFT), hypervalent iodine, iodine(III)

## Abstract

The reaction mechanism for the chlorination and bromination of 2-naphthol with PIDA or PIFA and AlX_3_ (X = Cl, Br), previously reported by our group, was elucidated via quantum chemical calculations using density functional theory. The chlorination mechanism using PIFA and AlCl_3_ demonstrated a better experimental and theoretical yield compared to using PIDA. Additionally, the lowest-energy chlorinating species was characterized by an equilibrium of Cl–I(Ph)–OTFA–AlCl_3_ and [Cl–I(Ph)][OTFA–AlCl_3_], rather than PhICl_2_ being the active species. On the other hand, bromination using PIDA and AlBr_3_ was more efficient, wherein the intermediate Br–I(Ph)–OAc–AlBr_3_ was formed as active brominating species. Similarly, PhIBr_2_ was higher in energy than our proposed species. The reaction mechanisms are described in detail in this work and were found to be in excellent agreement with the experimental yield. These initial results confirmed that our proposed mechanism was energetically favored and therefore more plausible compared to halogenation via PhIX_2_.

## Introduction

**Scheme 1:**
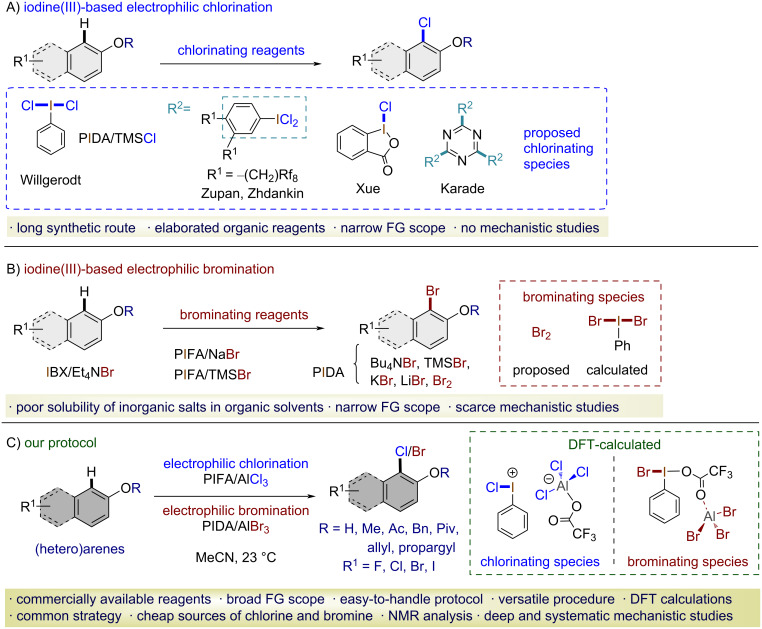
Representative protocols for the oxidative aromatic chlorination and bromination with iodine(III) reagents.

Hypervalent iodine(III) reagents have gained attention as strong oxidants with a low toxicity [1–8] and due to the ability to mimic reactivity [9] usually associated with transition metals [10–11]. Iodine(III) compounds have been used for the formation of different bond types, such as C–C [12–13], C–O [14–15], C–N [16], C–S [17], C–CN [18], C–F [19–21], C–I [22–23], C–NO_2_ [24–25] and, in the context of this work, C–X (X = Cl, Br) [26–31]. So far, different protocols for the halogenation of arenes using iodine(III) reagents have been described, mainly using (diacetoxyiodo)benzene (PIDA)/TMSCl, PIDA/TMSBr [32], and [bis(trifluoroacetoxy)iodo]benzene (PIFA)/TMSBr [33]. We have recently developed a new protocol for the oxidative chlorination and bromination of naphthols using the PIFA/AlCl_3_ [26] and PIDA/AlBr_3_ [28–29] systems. These unprecedented protocols combined iodine(III) reagents and aluminum salts to achieve chlorination and bromination of electron-rich arenes under mild and experimentally straightforward conditions (Scheme 1).

**Scheme 2:**
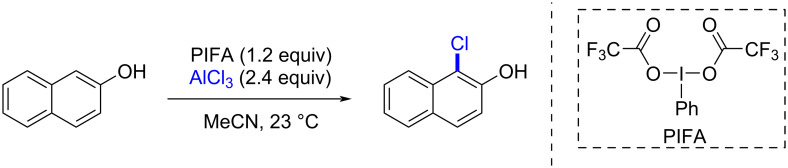
Chlorination of 2-naphthol using the PIFA/AlCl_3_, 1:2 system.

The synthesis of aryl halides is of great academic and industrial importance. Recently, our research group has developed a new procedure for the *ortho*-selective chlorination of phenols under mild conditions in a short reaction time [26]. The chlorinating species was generated in situ simply by mixing PIFA with a Lewis acid, in this case AlCl_3_. The importance of this protocol arises from the oxidation of an AlCl_3_-based chlorine atom_,_ which is an available and cheap reagent. Then it is used as an electrophile source in the chlorination process with an umpolung reactivity. In contrast to the suggested traceroute where the chlorine or bromine atom is attached to the hypervalent iodine center of the plausible reagent PhIX_2_ (X = Cl, Br), our new protocol opens up a broad path for the reaction through different halogenating species. For a deeper understanding of these reactions, we explored different pathways of the reaction mechanisms for the *ortho*-halogenation using 2-naphthol as a model substrate (Scheme 2). In such a way, we found a reaction pathway that was energetically favored.

**Scheme 3:**
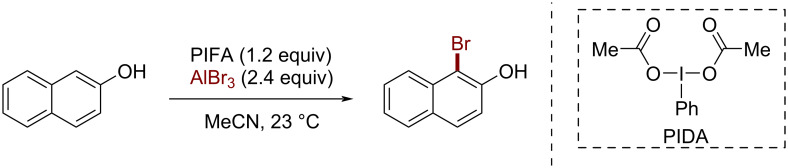
Bromination of 2-naphthol using the PIDA/AlBr_3_, 1:2 system.

Based on our successful procedure for chlorination, we also developed an efficient protocol for the electrophilic bromination of arenes, mainly phenols [28–29]. Accordingly, the bromination reaction was initially explored by mixing PIFA and AlBr_3_, which gave an acceptable yield (84%). However, other iodine(III) reagents were tested as oxidants during the optimization process. Thus, when mixing PIDA with aluminum bromide, the reaction occurred with an unexpectedly higher yield (93%) than with PIFA. Therefore, the bromination reaction proceeded in the presence of PIDA/AlBr_3_ as a brominating system using MeCN as solvent (Scheme 3).

In light of the relevance of this newly discovered reactivity and the scarce mechanistic and theoretical studies available [33], we computationally explored all of the different plausible pathways to elucidate the most feasible route that allowed the reported halogenation under these new reaction conditions. In this work, we systematically investigated the influence of PIDA and PIFA in the chlorination and bromination reactions. Interestingly, we found an excellent agreement between the theoretical predictions and the experimental results.

## Results and Discussion

### Computational details

The equilibrium geometry of reagents and products, the stationary points, and transition-state structures were optimized by density functional theory (DFT) calculations employing the software Gaussian 16 [34]. Although the B3LYP functional could be suitable for these calculations, e.g., for tracing reaction pathways, nitrations, halogenations, or FC acylations in solution, we found the ω-B97XD functional [35] appropriate for this study because it considered dispersion interactions through a range separation (22% for short range and 100% Hartree–Fock for long range), which properly describes thermochemistry and noncovalent interactions. When searching for the critical points along the potential surface energy of the possible chlorination and bromination pathways studied in this work, Br and I atoms were treated with the revised version of the LANL2DZ basis set and effective core potential, referred to as LANL08(d), providing d-type polarization functions. Meanwhile, the 6-31G(d) basis set was used for the other atoms (i.e., H, C, O, F, Al, etc.).

Geometry optimizations were carried out without any symmetry constraints, and the stationary points were characterized by analytical frequency calculations, i.e., energy minima (reactants, intermediates, and products) must exhibit only positive harmonic frequencies, whereas each energy maximum (transition state) exhibited only one negative frequency. From these last calculations, zero-point energy, thermal, and entropy corrections were obtained, which were added to the electronic energy to express the calculated values as Gibbs free energy at 298 K and 1 atm.

All our calculations were performed in the gas phase. Then, the solvent effects were included according to the polarizable continuum model via the solvent model density (SMD) option considering Truhlar’s model [36–40] and MeCN as the solvent. Single-point calculations were improved using a mixed basis set of triple-ζ quality with a polarization function, 6-311G(d,p) for all atoms except for Br and I, which were treated with the LANL08d relativistic pseudopotential [41–43], i.e., the composite level of theory used is the following: (SMD: MeCN) ω-B97XD/(6-311G(d,p),LANL08d)//ω-B97XD/6-31G(d), LANL08d.

### Chlorination mechanism

**Scheme 4:**
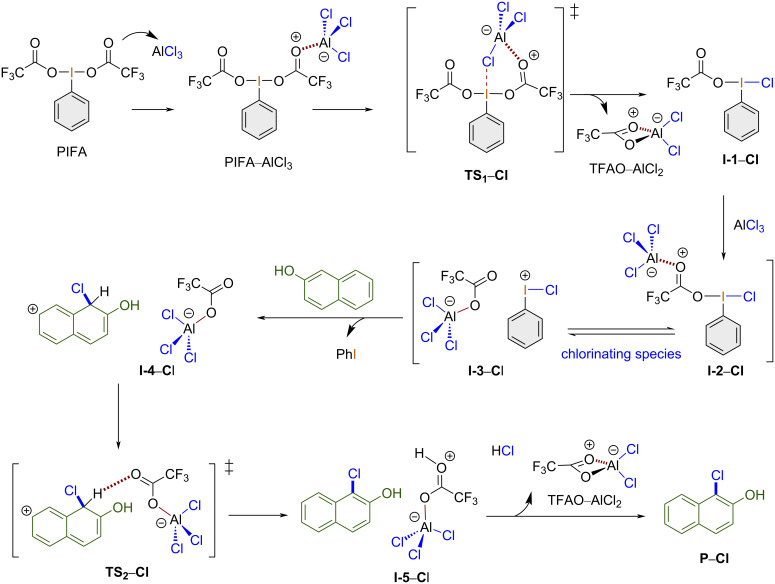
Reaction mechanism for the chlorination of 2-naphthol using the PIFA/AlCl_3_, 1:2 system.

The reaction mechanism for the chlorination of 2-naphthol using one equivalent of PIFA and two equivalents of aluminum chloride is outlined in Scheme 4.

The chlorination mechanism starts when PIFA coordinates the first equivalent of aluminum chloride to give the corresponding adduct PIFA–AlCl_3_. Next, a chlorine atom is transferred to the iodine(III) center to yield **I-1–Cl** via **TS_1_–Cl** with the release of the complex TFAO–AlCl_2_. Then, the second equivalent of aluminum chloride coordinates the TFAO ligand, giving rise to the chlorinating species **I-2–Cl** in equilibrium with **I-3–Cl**. At this point, 2-naphthol reacts, leading to the formation of the ion pair **I-4–Cl** via chlorine atom transfer, which then yields the adduct **I-5–Cl** trough transition state **TS_2_–Cl**. Then, the release of the second equivalent of the TFAO–AlCl_2_ complex yields the final product 1-chloro-2-naphthol (**P–Cl)**.

**Figure 1:**
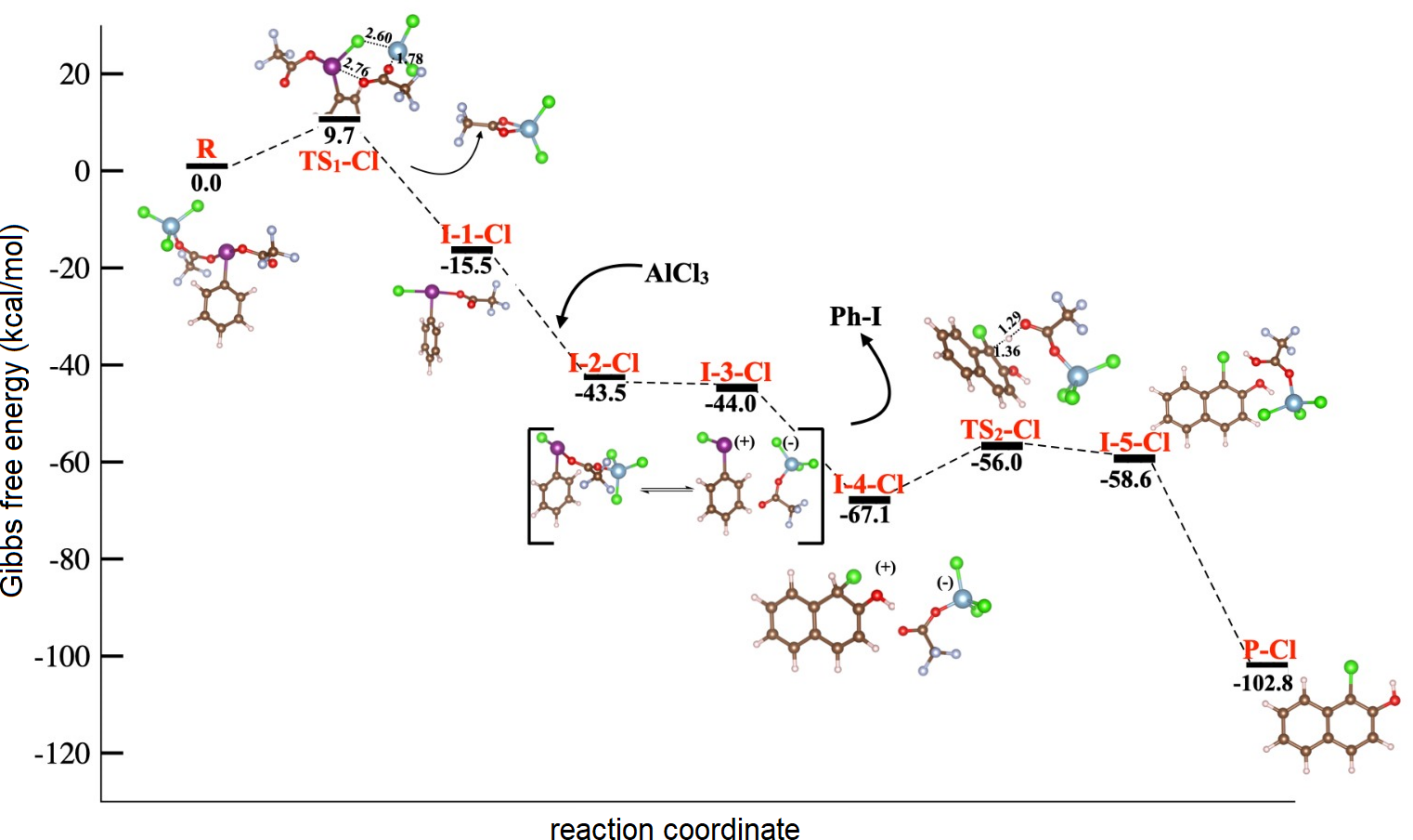
Energy profile for the chlorination of 2-naphthol in the presence of PIFA and AlCl_3_.

The calculated mechanism for the chlorination reaction starts with coordination of a PIFA oxygen atom to aluminum chloride. This generates a highly exergonic PIFA–AlCl_3_ adduct. In Figure 1, the Gibbs free energy of this adduct is set as 0 kcal/mol for more clarity. Herein, one chlorine atom is transferred from aluminum to the hypervalent iodine(III) center through six-membered-ring transition state **TS_1_–Cl** (Δ*G*^‡^ = 9.7 kcal/mol, selected bond lengths 2.76, 1.22, 1.27, 1.78, 2.60, and 2.86 Å for I–O, O–C, C–O, O–Al, Al–Cl, and Cl–I, respectively). Then, the tetracoordinate TFAO–AlCl_2_ salt is released, giving rise to intermediate **I-1–Cl** (Δ*G* = −25.2 kcal/mol), which contains the key Cl–I(III) bond, in a formal TFAO/Cl ligand exchange. The Cl–I bond length is 2.46 Å, with the halogen atom sharing the hypervalent iodine bond in the equatorial position. Next, the second equivalent of aluminum chloride coordinates to the TFAO ligand, forming active chlorinating species **I-2–Cl** (Δ*G* = −18.3 kcal/mol). This energetically favored step is in equilibrium with the ion pair **I-3–Cl** (Δ*G* = −0.5 kcal/mol). It is worth mentioning that the slight difference in energy between both states indicates the importance of the spontaneous interconversion of both species, which is observed only in the presence of two equivalents of the Lewis acid.

After the addition of 2-naphthol, the chlorine atom is introduced barrier-free into the phenolic ring, producing the nonaromatic intermediate **I-4–Cl** (Δ*G* = −23.1 kcal/mol). Next, aromatization assisted by TFAO–AlCl_2_ via **TS_2_–Cl** (Δ*G*^‡^ = 11.1 kcal/mol) and hydrogen transfer from the nonaromatic intermediate to TFAO–AlCl_2_ are observed. In **TS_2_–Cl**, the energy barrier must be overcome to give rise to the 1-chloro-2-naphthol adduct with TFA–OH–AlCl_2_, **I-5–Cl** (Δ*G* = −2.6 kcal/mol), which spontaneously yields the final 1-chloro-2-naphthol (**P–Cl**) with concomitant release of TFAO–AlCl_2_ in a highly exothermic process (Δ*G* = −44.2 kcal/mol, Figure 1).

Other relevant routes for this chlorination process, which involve a different stoichiometry or the formation of PhICl_2_ as chlorinating species, were also investigated and ruled out. Thus, for the chlorination of 2-naphthol with the PIFA/AlCl_3_, 1:1 system, we found that in general, once the intermediate **I-1–Cl** is formed, the following coordination of 2-naphthol with the TFAO ligand via **TS_2_** is energetically less favored (Δ*G*^‡^ = 16.2 kcal/mol, see Supporting Information File 1 for details of the explored chlorination and bromination mechanisms). Additionally, for this mechanism, we identified that the formation of **TS_4_** has the highest energy barrier (Δ*G*^‡^ = 20.2 kcal/mol), becoming a less probable route. This result also confirms the relevance of using two equivalents of aluminum chloride.

The aromatic chlorination with iodine(III) reagents broadly employs PhICl_2_ [7]. Thus, we explored two alternatives for the chlorination of 2-naphthol to identify or rule out this potential reaction pathway. The first explored mechanism involves PIFA/AlCl_3_ and the second PIDA/AlCl_3_ (see Figures S2 and S3, respectively, Supporting Information File 1). In both cases, the route involves the formation of PhICl_2_ as the chlorinating reagent by considering two equivalents of AlCl_3_ (PIFA/AlCl_3_ or PIDA/AlCl_3_, 1:2). Overall, we characterized four transition states along the reaction coordinates for both pathways. Although the PIFA-assisted mechanism follows a similar route to that described in Figure 1 until the formation of the active chlorinating species, in this case, the formation of **TS_2_** requires 18.1 kcal/mol, which is an energetically more demanding process than the equilibration between **I-2–Cl** and ion pair **I-3–Cl**, proposed as active chlorinating species in Figure 1 and requiring less than 1 kcal/mol. It is worth mentioning that once PhICl_2_ is formed, the energy barrier to **TS_3_** is 21.5 kcal/mol. These energy differences suggest that the traceroute PhICl_2_ is less viable for the chlorination of 2-naphthol.

On the other hand, in the presence of PIDA (Figure S3, Supporting Information File 1), when the reaction occurs through the chlorinating species PhICl_2_, we found that **TS_1_**, **TS_2_**, and **TS_4_** require 17.7, 13.8, and 16.5 kcal/mol, respectively. Considering the high transition-state energy barrier in the proposed mechanism shown in Figure 1 for **TS_2_–Cl** (11.1 kcal/mol), this route is less probable. Additionally, we observed that chlorination of naphthol (the formation of **I-6**) could be the determining step since we found a coupling between the ring of the chlorinating species and naphthol during **TS_4_**, i.e., it could disfavor the PIDA-assisted chlorination traceroute via PhICl_2_. Thus, using PIFA and two equivalents of AlCl_3_ resulted in the highest yield, which is in agreement with our experiments.

As a consequence of the previous analysis, the chlorination process is energetically favored in the presence of PIFA/AlCl_3_, 1:2 through the formation of PhICl–TFAO–AlCl_3_ in equilibrium with [PhICl][TFAO–AlCl_3_] as chlorinating species.

### Bromination mechanism

**Scheme 5:**
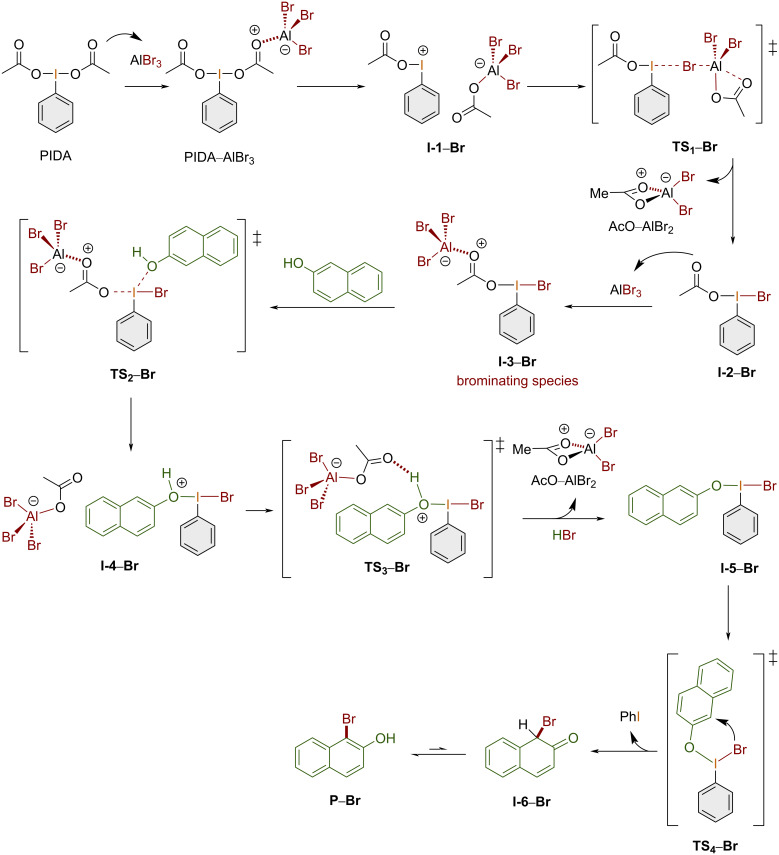
Calculated reaction mechanism for the bromination of 2-naphthol using the PIDA/AlBr_3_, 1:2 system.

The reaction mechanism for the bromination of 2-naphthol using one equivalent of PIDA and two equivalents of aluminum bromide is shown in Scheme 5.

PIDA coordinates the first equivalent of aluminum bromide to form the adduct PIDA–AlBr_3_, which spontaneously dissociates, giving ion pair **I-1–Br**. Next, via **TS_1_–Br**, complex AcO–AlBr_2_ is released with subsequent formation of intermediate **I-2–Br**. Then, the second equivalent of aluminum bromide coordinates to an acetate ligand, forming adduct **I-3–Br**, which is the brominating species. At this point, 2-naphthol reacts and forms **I-4–Br** via **TS_2_–Br**. Afterwards, the second equivalent of the AcO–AlBr_2_ complex and HBr are released with concomitant formation of **I-5–Br** trough **TS_2_–Br**. Then, **I-5–Br** spontaneously isomerizes to give **I-6–Br** via the transition state **TS_4_–Br**. Finally, **I-6–Br** tautomerizes, yielding the experimentally observed 1-bromo-2-naphthol (**P–Br**).

**Figure 2:**
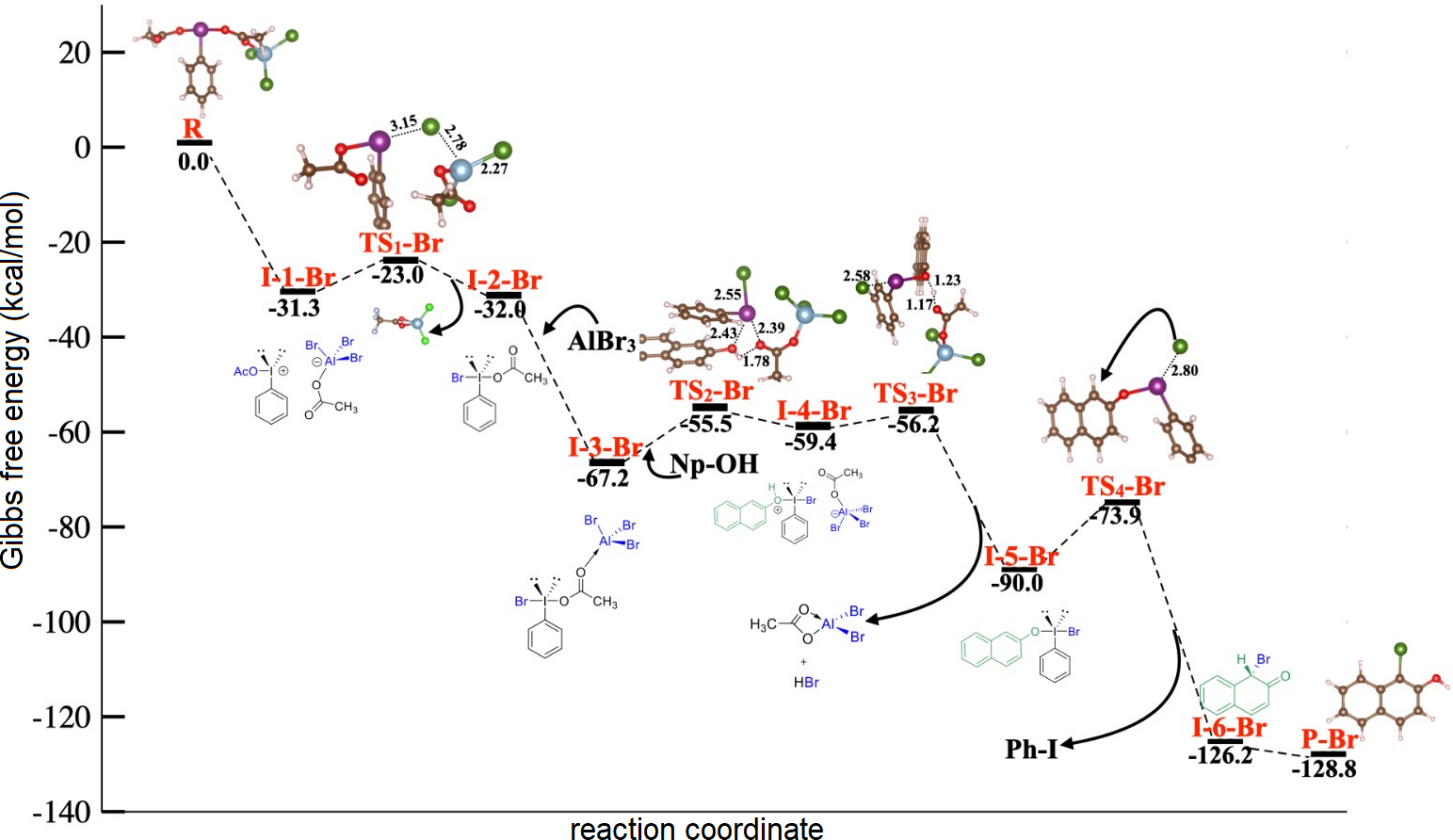
Calculated mechanism for the bromination of 2-naphthol in the presence of PIDA and AlBr_3_.

Based on our calculations, the bromination reaction proceeds through a stepwise mechanism. Thus, the reaction starts with the coordination of aluminum bromide to an acetate ligand in PIDA to form the PIDA–AlBr_3_ adduct in a highly exergonic process. Similar to the previous section, the Gibbs free energy at this point was set as 0 kcal/mol for reference. At this stage, the PIDA–AlBr_3_ adduct undergoes ionization, giving rise to the corresponding ion pair **I-1–Br** (Δ*G* = −31.3 kcal/mol) in a highly exergonic and energetically favorable process. Next, an intramolecular S_N_2 reaction of the formed aluminum anion transfers a bromine atom to the electrophilic iodine(III) center through **TS_1_–Br**, which has a feasible energy barrier of 8.3 kcal/mol. The I–Br and Br–Al bond lengths are 3.15 and 2.78 Å, respectively, and the I–Br–Al angle is 93.1^o^, which is close to the common T-shape of such hypervalent iodine(III) species. This step releases the tetracoordinate AcO–AlBr_2_ salt and gives rise to the intermediate **I-2–Br** (Δ*G* = −9 kcal/mol), which contains the key Br–I(III) bond with a length of 2.65 Å. Herein, we could identify an energetically favored AcO/Br ligand exchange that releases 35.2 kcal/mol. At this point, the second equivalent of aluminum bromide is coordinated by an acetate ligand to produce the active brominating species Br–I(Ph)–OAc–AlBr_3_ (**I-3–Br**). Then, 2-naphthol adds to the iodine(III) species to release the activated Br_3_Al–OAc ligand through transition state **TS_2_–Br** (Δ*G*^‡^ = 11.7 kcal/mol), which leads to the protonated intermediate **I-4–Br**. The next step is a deprotonation assisted by the released Br_3_Al–OAc species. This allows the formation of the AcO–AlBr_2_ salt via **TS_3_–Br** and the *trans* intermediate **I-5–Br**, which contains a Br–I(Ph)–O–naphthyl bond of 2.14 Å length. The last step is the bromination of **I-5–Br** by isomerization to the *cis* transition state **TS_4_–Br** (Δ*G*^‡^ = 16.1 kcal/mol), which yields the brominated nonaromatic intermediate **I-6–Br** in a highly exothermic step (Δ*G* = −52.3 kcal/mol). Finally, **I-6–Br** undergoes spontaneous aromatization, converting it into the experimentally observed 1-bromo-2-naphthol (**P–Br**), which is more stable than **I-6–Br** by 2.6 kcal/mol (Figure 2).

We also explored the plausible mechanisms for the bromination of 2-naphthol mediated by PIFA in a 1:2 ratio (Figure S4, Supporting Information File 1). In this proposal, we observed that the energy barriers to reach **TS_1_** (10.6 kcal/mol) and **TS_2_** (16.7 kcal/mol) are higher than those calculated for the mechanism shown in Figure 2, namely 8.3 kcal/mol for **TS_1_–Br** and 16.1 kcal/mol for **TS_4_–Br**.

Other possible mechanisms involve the formation of PhIBr_2_. For these scenarios, the reaction pathway with PIDA and PIFA, respectively, involves two equivalents of AlBr_3_ (Figures S5 and S6, Supporting Information File 1). Calculations indicated that each of these pathways proceeds along four transition states. Moreover, we found that the coordination of AlBr_3_ to **I-2** to form **TS_2_** has the highest energy barrier (determining step, Δ*G*^‡^ for **TS_2_** 53.3 kcal/mol) in the presence of PIDA. Meanwhile, formation of **TS_3_** (Δ*G*^‡^ = 21.3 kcal/mol) is the limiting step of the mechanism in the presence of PIFA.

Then, in the presence of the PIFA/AlBr_3_ system, bromination of 2-naphthol is the energetically most favored pathway. Although all these reactions occur through four transition states, significant energy differences exist concerning the PIDA/AlBr_3_ system. For example, the activation barrier of **TS_2_** was 41.6 kcal/mol higher in energy than that in the mechanism in Figure 2. A similar energy profile was obtained for the bromination of 2-naphthol in the presence of PIFA (and 2 equivalents of AlBr_3_) compared to Figure 2. The energy difference from **I-1** to **TS_1_** (2.3 kcal/mol for the reaction in the presence of PIFA) could be the reason why the experimental yield is higher in the presence of PIDA and two equivalents AlBr_3_ rather than PIFA and two equivalents AlBr_3_ when considering the different hypervalent iodine reagents for this reaction.

To find the correlations between experiments and theoretical calculations, chlorination and bromination of 2-naphthol using PIFA/AlCl_3_ and PIDA/AlBr_3_ were carried out. Consequently, we found an excellent correlation between the yield and the energy barrier (Table 1).

**Table 1:** Experimental yield by using different hypervalent iodine(III) reagents.

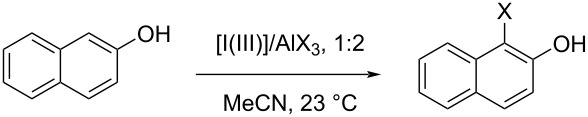			

reaction	PIFA	PIDA	AlX_3_

chlorination	63%	48%	AlCl_3_
bromination	84%	93%	AlBr_3_

## Conclusion

We elucidated the energetically most viable pathway for the chlorination and bromination of 2-naphthol using the novel systems PIFA/AlCl_3_ and PIDA/AlBr_3_ in a 2:1 ratio in both cases. We found that the energetically most favored reaction proceeds through the chlorinating species **I-2–Cl** and **I-3–Cl** (rather than PhICl_2_), which are in an equilibrium. The bromination is more efficient with PIDA/AlBr_3_ through the formation of the intermediate **I-3–Br** as active brominating species. Similarly, involvement of PhIBr_2_ is energetically less favored compared to our proposed pathway. One key step is the coordination of a second equivalent of AlX_3_ to TFAO or AcO in PIFA or PIDA to promote the formation of the active halogenating species **I-2** and **I-3** for chlorination and bromination, respectively. Although bromination reactions in the presence of PIDA and PIFA give an excellent experimental yield, slight energy differences in the pathways explained why PIFA/AlCl_3_ for chlorination and PIDA/AlBr_3_ for bromination are better choices for these reactions.

## Supporting Information

File 1 Optimized Cartesian coordinates of all structures and alternative mechanisms.

## Data Availability

Additional research data is not shared.
