# Supplementary material for: Divergent role of PIDA and PIFA in the AlX3 (X = Cl, Br) halogenation of 2-naphthol: a mechanistic study
Source: Beilstein J Org Chem. 2024 Jul 15;20:1580–9. doi: 10.3762/bjoc.20.141 (PMC11285080; doi:10.3762/bjoc.20.141)
Supplement: File 1 — Optimized Cartesian coordinates of all structures and alternative mechanisms. [file Beilstein_J_Org_Chem-20-1580-s001.pdf]

BEILSTEIN JOURNAL OF ORGANIC CHEMISTRY

Supporting Information

for

Divergent role of PIDA and PIFA in the AlX_3 (X = Cl, Br) halogenation of 2-naphthol: a mechanistic study

Kevin A. Juárez-Ornelas, Manuel Solís-Hernández, Pedro Navarro-Santos, J. Oscar C. Jiménez-Halla and César R. Solorio-Alvarado

Beilstein J. Org. Chem. **2024,** *20,* 1580–1589. doi:10.3762/bjoc.20.141

Optimized Cartesian coordinates of all structures and alternative mechanisms

License and Terms: This is a supporting information file under the terms of the Creative Commons Attribution License (https://creativecommons.org/licenses/by/4.0). Please note that the reuse, redistribution and reproduction in particular requires that the author(s) and source are credited and that individual graphics may be subject to special legal provisions.
The license is subject to the *Beilstein Journal of Organic Chemistry* terms and conditions: (https://www.beilstein-journals.org/bjoc/terms)

INDEX	PAGE
**1.** Computational details	**S1**
**2.** Reaction mechanism for the chlorination of 2-naphthol using the PIFA-$AlCl_3$ (1:2) system. Geometries of intermediates and transitions states.	**S2**
**3.** Reaction mechanism for the bromination of 2-naphthol using the PIDA-$AlCl_3$ (1:2) system. Geometries of intermediates and transitions states.	**S5**
**4.** Alternative mechanisms explored for the chlorination of 2-naphtol	
a. The PIFA-$AlCl_3$ (1:1) system	**S11**
b. The PIFA-$AlCl_3$ (1:2) system via the formation of $PhICl_2$	**S13**
c. The PIDA-$AlCl_3$ (1:2) system via the formation of $PhICl_2$	**S15**
**5.** Alternative mechanisms explored for the bromination of 2-naphtol	
a. The PIFA-$AlBr_3$ (1:2) system	**S18**
b. The PIFA-$AlBr_3$ (1:2) system via the formation of $PhIBr_2$	**S21**
c. The PIDA-$AlBr_3$ (1:2) system via the formation of $PhIBr_2$	**S24**

1. Computational details

The geometric optimization of reagents, adducts, transition states, and products was carried out employing the Gaussian 161 software at the gas phase. The potential energy surface was explored by using DFT calculations at the level of theory ω-B97XD/(6-311G(d,p),LANL08d)//ω-B97XD/6-31G(d). Once the critical points were found, the solvent effects were considered by performing energy calculations over each geometry using the polarizable continuum model (PCM) using the SMD proposed model of Truhlar and coworkers2. The geometries of the critical points of the proposed mechanisms are the following:

2. Reaction mechanism for the chlorination of 2-naphthol using the PIFA-$AlCl_3$ (1:2) system. Geometries of intermediates and transition states.

Reagents

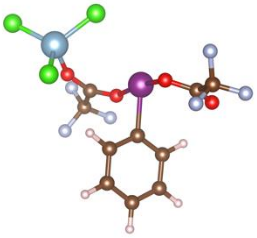

I	-0.715876	-0.166764	-0.538108
C	-1.140209	1.555499	0.561082
C	-0.766600	1.570977	1.899351
C	-1.759824	2.623374	-0.075213
C	-1.036188	2.721874	2.629104
H	-0.277442	0.721290	2.361566
C	-2.016728	3.760240	0.678462
H	-2.046658	2.567005	-1.116950
C	-1.656011	3.807506	2.021096
H	-0.757162	2.764636	3.675395
H	-2.500769	4.609976	0.211430
H	-1.860619	4.701650	2.599260
O	3.006426	-0.042313	-0.092155
C	2.423303	0.886642	-0.684759
O	1.223533	1.005913	-0.988438
C	3.334480	2.083058	-1.061627
F	3.616245	2.766678	0.050565
F	4.468417	1.651033	-1.597470
F	2.736673	2.898461	-1.919193
O	-3.644695	0.406992	-1.415822
C	-3.606185	-0.509040	-0.650179
O	-2.536362	-1.013560	-0.058681
C	-4.869255	-1.280262	-0.191760
F	-4.743266	-2.579965	-0.449764
F	-5.938139	-0.820118	-0.822479
F	-5.043994	-1.120102	1.121006
Al	2.780476	-1.774238	0.543400
Cl	1.697535	-2.708838	-1.055279
Cl	4.702110	-2.545019	0.884673
Cl	1.573446	-1.499104	2.286387

TS1-Cl

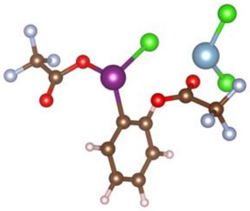

I	0.867926	-0.159466	-1.021628
C	0.694023	-1.483450	0.581382
C	0.217869	-1.017159	1.799471
C	1.012398	-2.816849	0.344727
C	0.043568	-1.942112	2.820456
H	0.009598	0.031669	1.964762
C	0.820363	-3.720356	1.379397
H	1.407531	-3.139729	-0.609496
C	0.334847	-3.284277	2.608468
H	-0.330675	-1.601509	3.778661
H	1.055962	-4.766476	1.222785
H	0.187757	-3.999338	3.410234
O	-2.313161	0.208681	-0.182512
C	-2.453519	-0.763364	-0.994159
O	-1.570775	-1.297069	-1.640042
C	-3.899886	-1.303442	-1.086975
F	-4.224307	-1.879974	0.074207
F	-4.750336	-0.302215	-1.310876
F	-4.024658	-2.197042	-2.055060
O	3.778498	-1.244551	-0.701749
C	3.709395	-0.078956	-0.473453
O	2.622284	0.692298	-0.488854
C	4.933690	0.794107	-0.090396
F	5.104354	1.759652	-0.991887
F	6.023027	0.042866	-0.056945
F	4.753584	1.347193	1.104807
Al	-2.301016	1.768595	0.670258
Cl	0.261620	2.178457	0.502380
Cl	-3.285943	3.254403	-0.410357
Cl	-2.679402	1.438983	2.705595

I-1-Cl

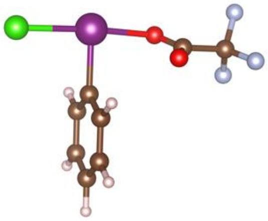

I	0.852585	-1.184867	-0.109129
C	1.191965	0.884745	-0.062962

C	1.569170	1.515612	-1.239357
C	1.019383	1.555982	1.137760
C	1.785888	2.886927	-1.201403
H	1.695957	0.962108	-2.161467
C	1.242249	2.927528	1.149535
H	0.699991	1.036390	2.030850
C	1.623616	3.587812	-0.011968
H	2.081221	3.404307	-2.107002
H	1.111348	3.476550	2.074873
H	1.794016	4.658479	0.008927
O	-1.214276	-0.690183	-0.577445
C	-1.952988	-0.245541	0.394579
O	-1.636009	-0.040817	1.537541
C	-3.395396	0.029854	-0.099752
F	-3.388786	0.969760	-1.052173
F	-3.939851	-1.077576	-0.613712
F	-4.168012	0.451323	0.893888
Cl	3.247016	-1.578880	0.282721

I-2-Cl

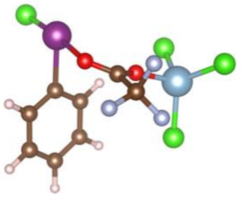

I	2.027269	-0.658431	-0.918673
C	1.875503	0.244562	0.968834
C	1.275935	1.491476	1.049061
C	2.337251	-0.463836	2.070291
C	1.134618	2.054713	2.311096
H	0.913577	2.009709	0.169349
C	2.182532	0.123731	3.318337
H	2.795439	-1.439635	1.968420
C	1.583280	1.373940	3.435754
H	0.647358	3.017356	2.405426
H	2.529547	-0.402223	4.200199
H	1.458043	1.818968	4.416210
O	-1.133595	-0.242164	-0.306512
C	-0.881373	-1.425882	-0.008280
O	0.233160	-1.965388	-0.129053
C	-2.020813	-2.329147	0.527787
F	-2.938965	-1.603334	1.150489
F	-2.587207	-2.957914	-0.500998
F	-1.543317	-3.234106	1.373209
Cl	3.942469	0.614603	-1.554066
Al	-2.258618	1.231679	-0.493219
Cl	-4.015448	0.532184	-1.429718
Cl	-2.454652	1.983380	1.482797
Cl	-1.032364	2.474664	-1.723433

I-3-Cl (cation)

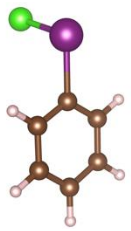

I	2.045080	-0.544362	-0.932900
C	1.849314	0.285537	0.957253
C	1.212787	1.522985	1.070145
C	2.351464	-0.430513	2.045543
C	1.076734	2.055656	2.342357
H	0.837028	2.056360	0.205417
C	2.198486	0.131428	3.303142
H	2.844324	-1.387572	1.924856
C	1.567058	1.364408	3.447618
H	0.587914	3.014215	2.467647
H	2.576178	-0.396345	4.170480
H	1.454682	1.792891	4.436848
Cl	4.101772	0.320243	-1.598168

I-3-Cl (anion)

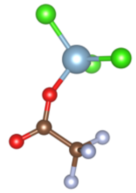

O	-1.097329	-0.287438	-0.413100
C	-0.794318	-1.457448	0.030496
O	0.295522	-1.961748	0.066106
C	-2.012293	-2.294438	0.511133
F	-2.907286	-1.560446	1.181417
F	-2.641185	-2.840921	-0.543063
F	-1.632264	-3.294603	1.312070
Al	-2.174212	1.129173	-0.511512
Cl	-4.052609	0.546719	-1.381173
Cl	-2.428989	1.926602	1.469360
Cl	-1.188230	2.558521	-1.778665

I-4-Cl (cation)

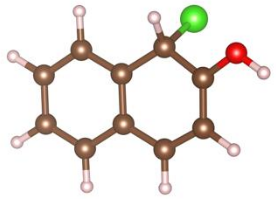

Cl	1.491701	-1.665900	0.852624
C	-2.825305	-1.045598	-0.419419
C	-1.468078	-1.297089	-0.620586
C	-0.539203	-0.316673	-0.331131
C	-0.973723	0.932295	0.160701

C	-2.344306	1.168715	0.366019
C	-3.265300	0.179160	0.078484
C	0.929591	-0.579924	-0.484648
C	-0.019178	1.958882	0.393415
C	1.803220	0.634196	-0.378810
H	1.156781	-1.106158	-1.413041
H	-3.547738	-1.818830	-0.655623
H	-1.147181	-2.263427	-0.995129
H	-2.672842	2.131592	0.743344
H	-4.322793	0.355291	0.233204
H	-0.378931	2.908698	0.780457
C	1.329283	1.837718	0.122337
H	2.000473	2.676871	0.270241
O	3.022647	0.427744	-0.767454
H	3.610122	1.184229	-0.636596

I-4-Cl (anion)

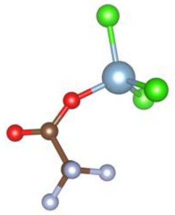

O	-1.097329	-0.287438	-0.413100
C	-0.794318	-1.457448	0.030496
O	0.295522	-1.961748	0.066106
C	-2.012293	-2.294438	0.511133
F	-2.907286	-1.560446	1.181417
F	-2.641185	-2.840921	-0.543063
F	-1.632264	-3.294603	1.312070
Al	-2.174212	1.129173	-0.511512
Cl	-4.052609	0.546719	-1.381173
Cl	-2.428989	1.926602	1.469360
Cl	-1.188230	2.558521	-1.778665

TS2-Cl

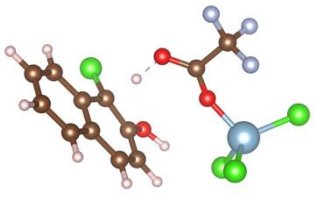

O	1.228023	0.201738	-0.429031
C	0.623347	1.296799	-0.538860
O	-0.572370	1.429136	-0.831059
C	1.429970	2.600198	-0.316478
F	2.283367	2.762999	-1.324403
F	0.634134	3.654705	-0.251051
F	2.117037	2.508666	0.820956
Cl	-2.641515	-0.543891	-2.718478
Al	2.624709	-0.724788	0.358952
Cl	4.464979	0.153428	-0.150008
Cl	2.091818	-0.758599	2.414495
Cl	2.373773	-2.680380	-0.531502
C	-4.737966	0.807613	2.054905
C	-3.653472	0.023929	2.363250
C	-2.831784	-0.491315	1.340939
C	-3.123299	-0.191252	-0.009550
C	-4.230075	0.616067	-0.304455
C	-5.024659	1.097779	0.713199
H	-1.502225	-1.550068	2.682323
H	-5.370877	1.200722	2.842083
H	-3.416852	-0.209090	3.396386
C	-1.725331	-1.339414	1.641159
C	-2.179778	-0.653401	-1.025184
H	-4.461817	0.854992	-1.334348
H	-5.883912	1.713084	0.469700
C	-1.212117	-1.638425	-0.687921
C	-0.947639	-1.903706	0.678723
H	-1.276575	0.351687	-0.913259
H	-0.134572	-2.569170	0.942194
O	-0.522110	-2.178536	-1.656179
H	0.309295	-2.574690	-1.335799

I-5-Cl

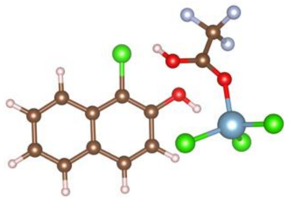

O	-2.465281	0.342861	-0.433286
C	-1.677642	1.259851	-0.605200
O	-0.657865	1.177527	-1.380109
C	-2.045101	2.617193	0.046768
F	-2.264795	2.469506	1.336800
F	-1.074566	3.510347	-0.144814
F	-3.150053	3.063340	-0.540543
Cl	1.910825	2.406771	-0.056046
Al	-2.535644	-1.561977	-0.261858
Cl	-4.433340	-2.057224	-1.003924
Cl	-0.851348	-2.303066	-1.290962
Cl	-2.304795	-1.661957	1.867237
C	5.787370	-1.249820	-0.640844
C	4.799736	-1.910871	0.035383
C	3.590236	-1.259248	0.377093
C	3.405047	0.099988	0.005383
C	4.445483	0.760356	-0.693813
C	5.603521	0.100117	-1.005120
H	2.703526	-2.970367	1.356442

H	6.710554	-1.757590	-0.897114
H	4.928461	-2.950365	0.320570
C	2.562056	-1.930212	1.083015
C	2.177907	0.720802	0.361894
H	4.322878	1.797657	-0.979272
H	6.389962	0.622622	-1.539207
C	1.195720	0.052531	1.048906
C	1.400951	-1.299017	1.410912
H	-0.006051	1.890680	-1.238649
H	0.612897	-1.829571	1.934494
O	0.034870	0.690663	1.358412
H	-0.522440	0.118545	1.900338

Product

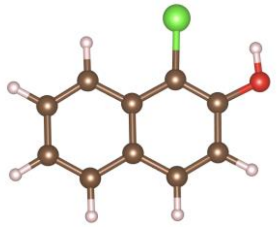

C	3.281358	-0.121724	-0.000046
C	2.533034	1.023815	-0.000125
C	1.118876	0.970694	-0.000095
C	0.473112	-0.295722	0.000019
C	1.272207	-1.465259	0.000098
C	2.638899	-1.376558	0.000067
H	0.830149	3.114482	-0.000263
H	4.364623	-0.068251	-0.000070
H	3.016247	1.996276	-0.000214
C	0.330671	2.150918	-0.000176
C	-0.947617	-0.313546	0.000043
H	0.792770	-2.436488	0.000186
H	3.233417	-2.284143	0.000129
C	-1.692822	0.841796	-0.000038
C	-1.029136	2.091863	-0.000150
H	-1.642481	2.985366	-0.000212
O	-3.038803	0.876993	-0.000021
H	-3.388876	-0.019404	0.000038
Cl	-1.809818	-1.843615	0.000176

3. Reaction mechanism for the bromination of 2-naphthol using the PIDA-$AlCl_3$ (1:2) system. Geometries of intermediates and transition states.

Reagents

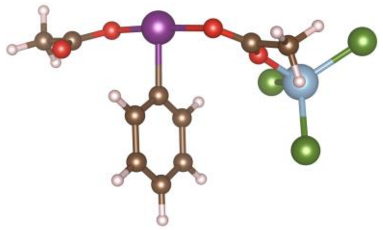

I	-2.263209	-1.078661	-0.570870
C	-2.004985	0.968363	-0.226948
C	-1.166625	1.355920	0.806902
C	-2.664751	1.865353	-1.056092
C	-0.969506	2.716756	1.004533
H	-0.663594	0.631866	1.435715
C	-2.450989	3.219348	-0.836415
H	-3.336757	1.523444	-1.832058
C	-1.604145	3.640615	0.183964
H	-0.296898	3.042702	1.788830
H	-2.951704	3.944965	-1.467189
H	-1.435951	4.700402	0.339236
O	0.882019	-0.582209	-0.690706
C	0.510137	-0.584914	-1.902107
O	-0.693305	-0.800166	-2.202692
C	1.487175	-0.329671	-3.004545
O	-5.128850	-0.076648	-0.381454
C	-4.882754	-0.654780	0.643882
O	-3.691776	-1.220866	0.906801
C	-5.834613	-0.840485	1.793802
Al	2.381537	-0.140038	0.291061
Br	1.723838	-0.574236	2.456837
Br	4.089899	-1.481158	-0.470007
Br	2.676693	2.109691	-0.151282
H	2.305621	-1.049589	-2.935420
H	1.001242	-0.394941	-3.974606
H	1.923136	0.663612	-2.866378
H	-5.986540	-1.904701	1.982505
H	-6.784027	-0.363263	1.560900
H	-5.407810	-0.405308	2.699124

I-1-Br (cation)

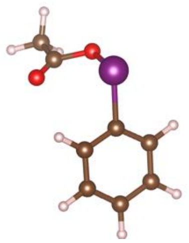

I	-0.610262	-1.131574	-0.204055
C	1.205207	-0.158329	-0.024878
C	1.574307	0.747217	-1.021273

C	2.009720	-0.465958	1.074377
C	2.811617	1.359701	-0.900886
H	0.913862	0.981175	-1.846655
C	3.240905	0.164513	1.164897
H	1.693305	-1.169690	1.834684
C	3.636977	1.067980	0.182541
H	3.129458	2.069085	-1.655376
H	3.891129	-0.052152	2.003862
H	4.602243	1.554325	0.263725
O	-1.601929	1.541072	-0.959859
C	-2.115977	1.286514	0.082208
O	-1.844436	0.099420	0.753307
C	-3.086537	2.097690	0.881287
H	-3.256187	3.041410	0.368127
H	-4.026404	1.553472	0.987576
H	-2.689913	2.275880	1.881773

I-1-Br (anion)

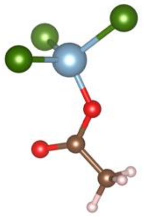

O	-1.307517	-0.032026	1.146309
C	-2.590953	-0.014221	0.857457
O	-3.056461	0.031643	-0.256281
C	-3.453626	-0.064595	2.104180
H	-3.270948	-1.002828	2.634457
H	-3.177060	0.747451	2.780441
H	-4.507645	0.009927	1.838568
Al	0.111202	-0.001305	0.065304
Br	0.175272	-1.896440	-1.299865
Br	0.193572	1.972242	-1.181246
Br	1.936566	-0.054705	1.538469

TS1-Br

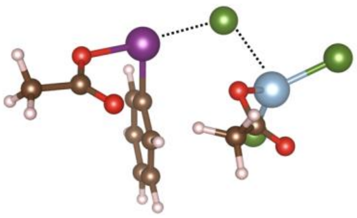

I	1.961573	-0.835124	-0.900981
C	1.668100	1.161101	-0.350960
C	1.562395	2.108924	-1.359877
C	1.575465	1.470528	0.999327
C	1.364445	3.430642	-0.986687
H	1.606212	1.830558	-2.405261
C	1.390278	2.801931	1.344079
H	1.654380	0.703176	1.755651
C	1.283096	3.774208	0.357604
H	1.261924	4.189303	-1.753528
H	1.303762	3.070131	2.390370
H	1.116492	4.808115	0.637465
O	-0.632536	-0.904713	1.132236
C	-0.886658	-0.761654	2.411009
O	-1.917681	-0.190223	2.748062
C	0.103693	-1.344110	3.371250
O	3.383066	-1.037081	1.809891
C	4.227358	-0.965113	0.957761
O	3.959112	-0.825758	-0.353154
C	5.713222	-1.008716	1.192297
Al	-2.110200	-0.150863	0.356121
Br	-0.949498	-0.754623	-2.101682
Br	-4.000596	-1.401659	0.179612
Br	-2.165812	2.123991	0.140435
H	1.119589	-1.286242	2.979609
H	0.024991	-0.849606	4.337911
H	-0.136236	-2.402576	3.506117
H	6.172547	-0.090810	0.821774
H	6.152289	-1.842108	0.641500
H	5.905928	-1.121169	2.256918

I-2-Br

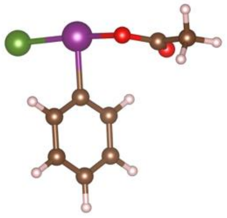

I	-0.174477	-0.954267	-0.218112
C	0.148754	1.119252	-0.105694
C	-0.188113	1.899273	-1.202323
C	0.680868	1.644891	1.061435
C	0.021155	3.269584	-1.118337
H	-0.613275	1.459305	-2.095936
C	0.882375	3.019100	1.120878
H	0.954676	1.002376	1.887231
C	0.553288	3.826353	0.039084
H	-0.236621	3.900618	-1.961471
H	1.299084	3.454565	2.021998
H	0.711639	4.897551	0.098512
Br	-2.746778	-0.537175	0.277849
O	2.503597	-0.845847	1.369149
C	2.782976	-1.067786	0.212124
O	1.898229	-1.146310	-0.768026
C	4.193553	-1.279049	-0.285219
H	4.272890	-2.246566	-0.784163
H	4.890529	-1.231494	0.549372
H	4.441817	-0.511509	-1.021009

I-3-Br

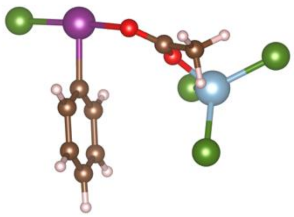

I	2.384800	-0.749225	0.792647
C	1.784464	1.181914	0.204222
C	1.215556	1.334704	-1.048615
C	1.925814	2.214956	1.117778
C	0.762900	2.599535	-1.399180
H	1.096482	0.501132	-1.728571
C	1.461744	3.469566	0.741538
H	2.369777	2.056435	2.092549
C	0.882792	3.658175	-0.507816
H	0.286256	2.739319	-2.361865
H	1.550361	4.297520	1.435484
H	0.507406	4.636805	-0.783745
O	-0.794038	-0.569885	0.792434
C	-0.484383	-0.456837	2.016344
O	0.717521	-0.529867	2.382150
C	-1.533630	-0.224725	3.056791
Al	-2.259260	-0.264468	-0.286362
Br	-1.497494	-0.786092	-2.397981
Br	-3.933465	-1.650639	0.472523
Br	-2.695542	1.985395	0.025894
H	-2.301755	-0.996835	2.979708
H	-1.094848	-0.215807	4.051277
H	-2.021846	0.732588	2.853319
Br	4.329494	-0.819230	-0.880687

TS2-Br

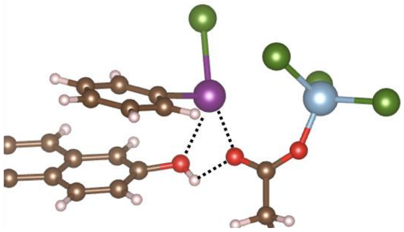

I	-0.271411	0.648683	-0.377844
C	-1.497555	0.066728	1.227569
C	-1.020030	-0.916040	2.080439
C	-2.749389	0.648860	1.378195
C	-1.852027	-1.350248	3.107514
H	-0.025040	-1.326083	1.962218
C	-3.560464	0.204492	2.411684
H	-3.093670	1.422777	0.704801
C	-3.115921	-0.797495	3.267851
H	-1.496798	-2.112858	3.791403
H	-4.545951	0.638226	2.536386
H	-3.757349	-1.142424	4.071442
O	0.441782	-1.627063	-0.633867
C	1.472232	-2.338248	-0.696292
O	2.655029	-1.923334	-0.531716
C	1.318781	-3.799761	-1.021256
Br	-0.140223	3.053040	0.463099
C	-7.903928	-0.228481	-0.345065
C	-7.034698	-1.203534	0.062070
C	-5.684579	-1.195397	-0.367979
C	-5.240715	-0.158249	-1.231611
C	-6.166140	0.835061	-1.640649
C	-7.463398	0.800776	-1.206982
H	-5.092955	-2.983411	0.699326
H	-8.935420	-0.243224	-0.010011
H	-7.370390	-1.998075	0.721949
C	-4.757647	-2.187927	0.041374
C	-3.886865	-0.135248	-1.647957
H	-5.829432	1.625127	-2.304826
H	-8.162331	1.566359	-1.526360
C	-3.022384	-1.094434	-1.203989
C	-3.454212	-2.142272	-0.360248
H	-3.525726	0.651827	-2.301663
H	-2.738707	-2.880153	-0.010128
O	-1.692330	-0.962837	-1.514370
H	-1.186030	-1.773377	-1.357128
H	1.224366	-3.901862	-2.106384
H	0.414258	-4.202383	-0.564639
H	2.193082	-4.362205	-0.700090
Al	3.590180	-0.401163	-0.123865
Br	2.824128	0.189379	1.982280
Br	3.006181	1.115345	-1.790892
Br	5.799009	-0.988877	-0.187594

I-4-Br (cation)

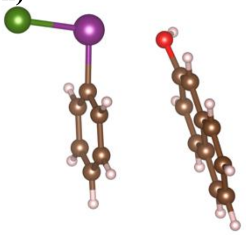

I	-1.954288	-0.683216	0.391674
C	-0.704664	0.963012	0.059542
C	-0.818622	2.061935	0.903431
C	0.244883	0.860615	-0.949822
C	0.074782	3.107375	0.715180
H	-1.577182	2.115121	1.674964
C	1.135478	1.913647	-1.102496
H	0.307696	-0.008046	-1.592000
C	1.049198	3.027335	-0.274854
H	0.008825	3.983436	1.349527
H	1.901316	1.853324	-1.866387
H	1.749601	3.844481	-0.403460
Br	-3.884532	0.032863	-1.044146
C	5.442196	0.007853	-1.293970
C	4.888260	0.559814	-0.171258

C	3.704661	0.018233	0.388759
C	3.102047	-1.111875	-0.227726
C	3.703431	-1.666563	-1.384908
C	4.843336	-1.116691	-1.905649
H	3.563105	1.431422	2.022057
H	6.348713	0.426920	-1.715823
H	5.352731	1.417167	0.305756
C	3.097324	0.577150	1.541730
C	1.905201	-1.639338	0.319087
H	3.251953	-2.536748	-1.851027
H	5.297812	-1.548413	-2.790327
C	1.344453	-1.045454	1.410486
C	1.932682	0.067865	2.043398
H	1.429815	-2.503756	-0.133470
H	1.458195	0.518492	2.909956
O	0.094419	-1.499838	1.832626
H	0.020096	-1.443930	2.790015

I-4-Br (anion)

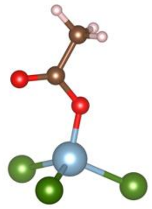

O	-1.307517	-0.032026	1.146309
C	-2.590953	-0.014221	0.857457
O	-3.056461	0.031643	-0.256281
C	-3.453626	-0.064595	2.104180
H	-3.270948	-1.002828	2.634457
H	-3.177060	0.747451	2.780441
H	-4.507645	0.009927	1.838568
Al	0.111202	-0.001305	0.065304
Br	0.175272	-1.896440	-1.299865
Br	0.193572	1.972242	-1.181246
Br	1.936566	-0.054705	1.538469

TS3-Br

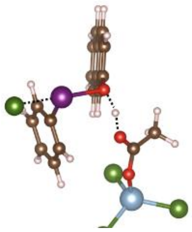

I	-3.312910	-1.169965	0.784849
C	-2.063635	-0.798769	-0.857253
C	-0.857326	-1.482846	-0.937804

C	-2.435465	0.205297	-1.740508
C	0.020706	-1.120941	-1.950512
H	-0.581220	-2.242615	-0.219466
C	-1.541948	0.540786	-2.750307
H	-3.377850	0.728975	-1.640109
C	-0.318575	-0.110768	-2.845378
H	0.978901	-1.622468	-2.025943
H	-1.798728	1.332509	-3.444225
H	0.389001	0.175625	-3.614679
O	0.039581	-0.849034	1.886981
C	1.188485	-0.352739	1.730564
O	2.043500	-1.016760	1.084273
C	1.546379	0.983446	2.306978
Br	-4.750184	-2.900606	-0.480455
C	-1.081715	5.901326	-1.209357
C	-1.999890	5.551901	-0.256301
C	-1.998150	4.251519	0.305180
C	-1.023829	3.315399	-0.131471
C	-0.085137	3.703756	-1.120178
C	-0.115966	4.966870	-1.646071
H	-3.684114	4.562127	1.626177
H	-1.090869	6.899411	-1.634032
H	-2.741891	6.269575	0.080619
C	-2.942279	3.846619	1.284882
C	-1.027029	2.009133	0.413469
H	0.661161	2.985590	-1.444769
H	0.609823	5.257565	-2.397917
C	-1.954017	1.652486	1.359443
C	-2.925268	2.582301	1.801435
H	-0.303360	1.289429	0.044637
H	-3.637990	2.270338	2.557023
O	-1.992764	0.385795	1.881313
H	-0.894455	-0.158225	2.003163
H	2.419063	0.861013	2.952938
H	0.723163	1.417653	2.871061
H	1.839038	1.664307	1.504106
Al	3.573187	-0.714197	0.119753
Br	5.232885	-0.239638	1.634696
Br	3.014831	1.119449	-1.212008
Br	3.833570	-2.619968	-1.134628

I-5-Br

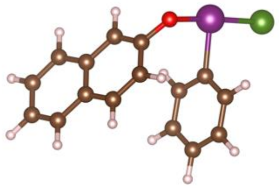

I	-1.130467	1.637360	-0.852914
C	-0.537830	0.998929	1.054520
C	-1.510886	0.774940	2.016767

C	0.800281	0.692581	1.255812
C	-1.117164	0.226017	3.230575
H	-2.549348	1.021709	1.832825
C	1.170237	0.130537	2.470275
H	1.538393	0.867376	0.484125
C	0.215225	-0.101982	3.451824
H	-1.858658	0.050047	4.001773
H	2.207413	-0.135226	2.638173
H	0.511040	-0.541603	4.397979
Br	-0.798485	4.174283	-0.089292
C	3.632352	-3.973419	0.282147
C	2.357495	-3.938245	0.779244
C	1.381870	-3.078722	0.217817
C	1.739734	-2.247425	-0.877878
C	3.067432	-2.310768	-1.374617
C	3.989281	-3.149244	-0.808577
H	-0.227846	-3.656010	1.545016
H	4.372314	-4.634232	0.721208
H	2.076711	-4.573274	1.614711
C	0.054211	-3.014662	0.714992
C	0.774150	-1.367307	-1.424149
H	3.343247	-1.679879	-2.214685
H	5.001386	-3.184793	-1.198383
C	-0.496188	-1.301105	-0.902336
C	-0.856179	-2.153990	0.175806
H	1.046188	-0.718633	-2.252426
H	-1.867562	-2.085376	0.560709
O	-1.417142	-0.420344	-1.349052

TS4-Br

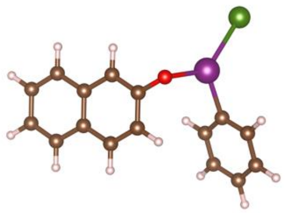

I	-1.766399	-0.546019	-2.555653
C	-2.942029	-1.006139	-0.884129
C	-4.275265	-1.371051	-1.057123
C	-2.330956	-1.012690	0.367617
C	-5.010436	-1.746674	0.058262
H	-4.730896	-1.361234	-2.040390
C	-3.079934	-1.399375	1.470943
H	-1.291052	-0.729173	0.479682
C	-4.412732	-1.763025	1.314817
H	-6.050310	-2.030762	-0.056151
H	-2.619159	-1.415103	2.452005
H	-4.993208	-2.062291	2.180685
Br	-1.797635	2.065318	-3.563109
C	4.053322	-4.417394	0.596885
C	2.704444	-4.631463	0.687092
C	1.815007	-4.062345	-0.256288
C	2.339911	-3.258643	-1.305071
C	3.742664	-3.056389	-1.372094
C	4.576793	-3.620771	-0.446063
H	0.007099	-4.886634	0.603472
H	4.726119	-4.858744	1.324512
H	2.298455	-5.245157	1.486221
C	0.411623	-4.268164	-0.192706
C	1.453669	-2.675502	-2.241851
H	4.145761	-2.445495	-2.174228
H	5.647853	-3.459889	-0.510591
C	0.099250	-2.904223	-2.167154
C	-0.422907	-3.711101	-1.117321
H	1.839501	-2.052269	-3.042578
H	-1.494645	-3.879438	-1.081395
O	-0.739357	-2.384481	-3.086239

I-6-Br

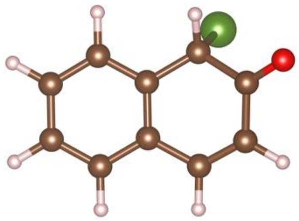

Br	1.555836	-1.620144	1.136768
C	-2.840058	-1.041955	-0.470293
C	-1.485001	-1.293335	-0.637490
C	-0.543559	-0.324508	-0.312092
C	-0.964840	0.918666	0.181459
C	-2.328614	1.157817	0.353196
C	-3.262502	0.184368	0.031188
C	0.919434	-0.597036	-0.459100
C	0.022585	1.957154	0.471955
C	1.820055	0.635555	-0.524835
H	1.149524	-1.251822	-1.296275
H	-3.566505	-1.804511	-0.728778
H	-1.152079	-2.256691	-1.011520
H	-2.656489	2.119628	0.735795
H	-4.320369	0.381218	0.166154
H	-0.342462	2.875623	0.924004
C	1.319808	1.837357	0.162666
H	2.031097	2.638731	0.324789
O	2.873258	0.601446	-1.112606

Product

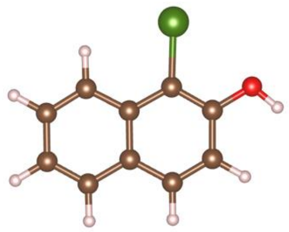

C	3.511048	-0.859391	-0.000241
C	3.134105	0.454330	-0.000328

C	1.765423	0.820195	-0.000241
C	0.768411	-0.195883	-0.000073
C	1.193438	-1.549314	0.000014
C	2.524374	-1.867740	-0.000061
H	2.130224	2.951394	-0.000436
H	4.561717	-1.128547	-0.000310
H	3.881928	1.241736	-0.000452
C	1.368465	2.178492	-0.000306
C	-0.598928	0.199277	0.000004
H	0.450149	-2.336341	0.000156
H	2.823610	-2.910644	0.000025
C	-0.957302	1.527127	-0.000044
C	0.049516	2.522336	-0.000215
H	-0.243616	3.569295	-0.000288
O	-2.266093	1.864422	0.000029
H	-2.356366	2.819031	0.000301
Br	-1.990577	-1.099939	0.000278

4. Alternative mechanisms explored for the chlorination of 2-naphthol

a. The PIFA-AlCl₃ (1:1) system

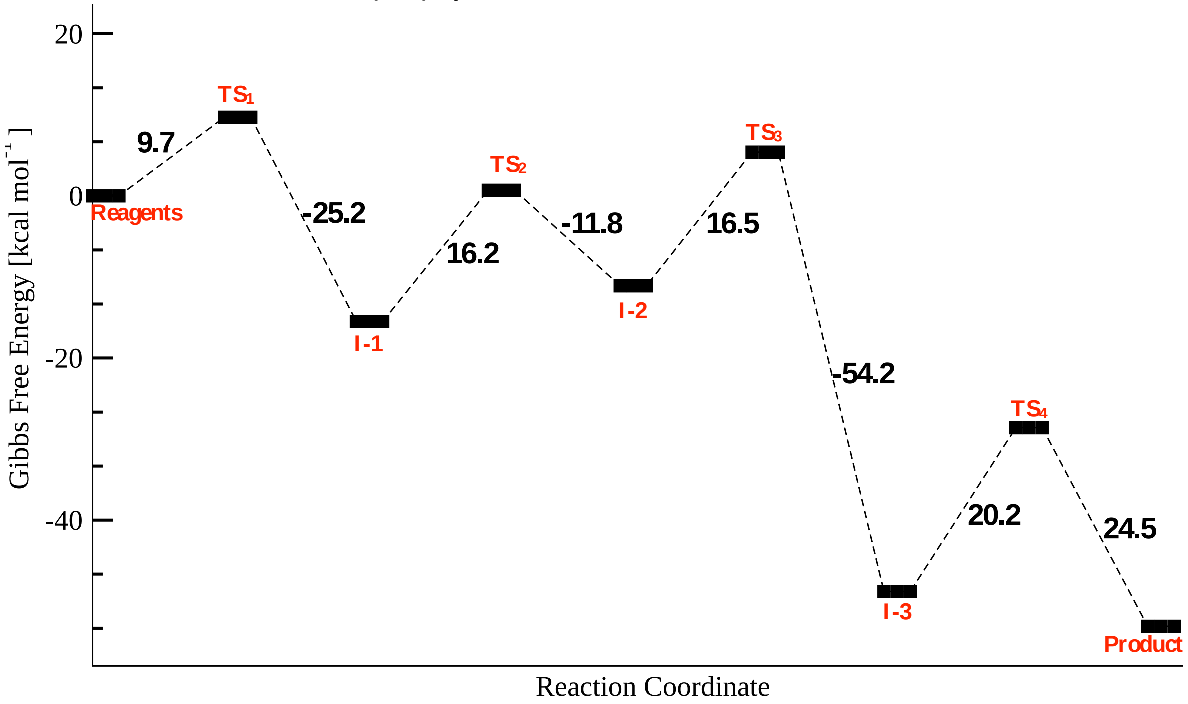

Figure S1. Energy profile for the chlorination of 2-naphthol in the presence of PIFA and $AlCl_3$ (1:1). Values represent the Gibbs free energy (in kcal/mol).

Table S1. Structures of intermediates and transition states with their corresponding Gibbs free energies (in kcal/mol) for the chlorination of 2-naphthol in the presence of PIFA- $AlCl_3$ (1:1).

Step	Intermediate (I) / Transition State (TS)	ΔG [kcal/mol]
Reagents	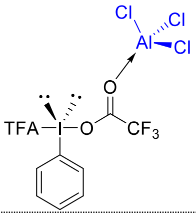	0.0
**TS_1**	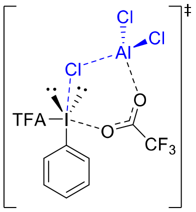	9.7
**I-1**	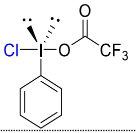	-15.5

**TS_2**	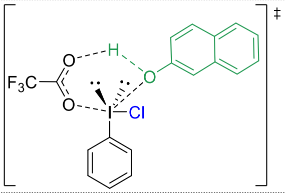	0.7
**I-2**	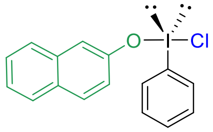	-11.1
**TS_3**	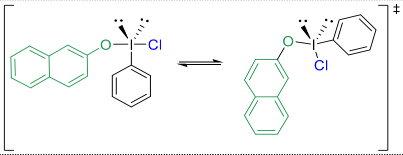	5.4
**I-3**	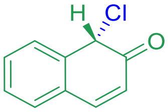	-48.8
**TS_4**	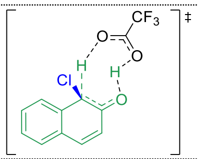	-28.6
Product	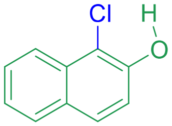	-53.1

4. Alternative mechanisms explored for the chlorination of 2-naphtol

b. The PIFA-$AlCl_3$ (1:2) system via the formation of $PhCl_2$

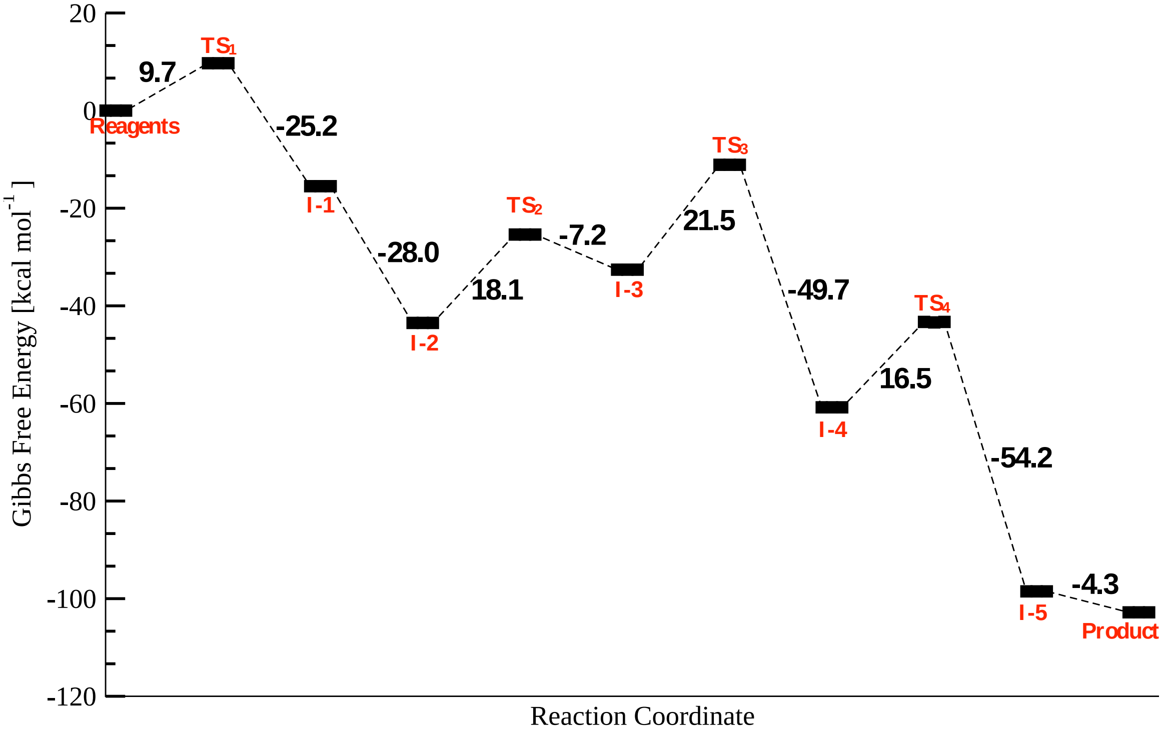

Figure S2. Energy profile for the chlorination of 2-naphthol in the presence of PIFA and $AlCl_3$ (1:2) via the formation of $PhICl_2$. Values represent the Gibbs free energy (in kcal/mol).

Table S2. Structures of intermediates and transition states with their corresponding Gibbs free energies (in kcal/mol) for the chlorination of 2-naphthol in the presence of PIFA - $AlCl_3$ (1:2) via the formation of $PhICl_2$.

Step	Intermediate (I) / Transition State (TS)	ΔG [kcal/mol]
Reagents	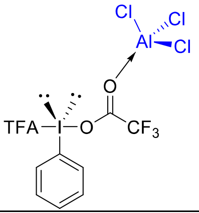	0.0
**TS_1**	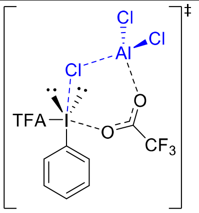	9.7

TFOAc-$AlCl_2$	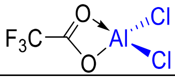	
**I-1**	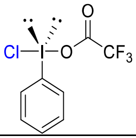	-15.5
**I-2**	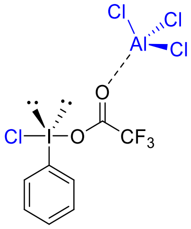	-43.5
**TS_2**	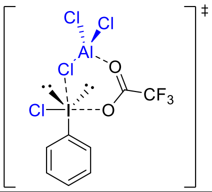	-25.4
**I-3**	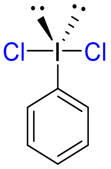	-32.6
**TS_3**	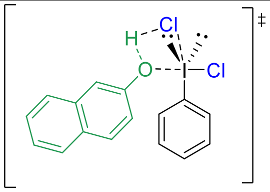	-11.1
**I-4**	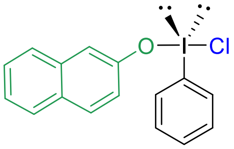	-60.8
**TS_4**	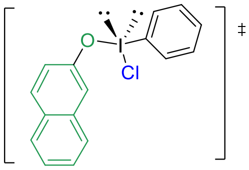	-44.3
**I-5**	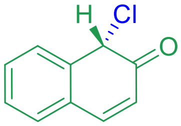	-98.5
Product	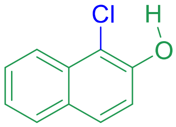	1-Cl-2-NpOH

4. Alternative mechanisms explored for the chlorination of 2-naphtol

c. The PIDA-$AlCl_3$ (1:2) system via the formation of $PhICl_2$

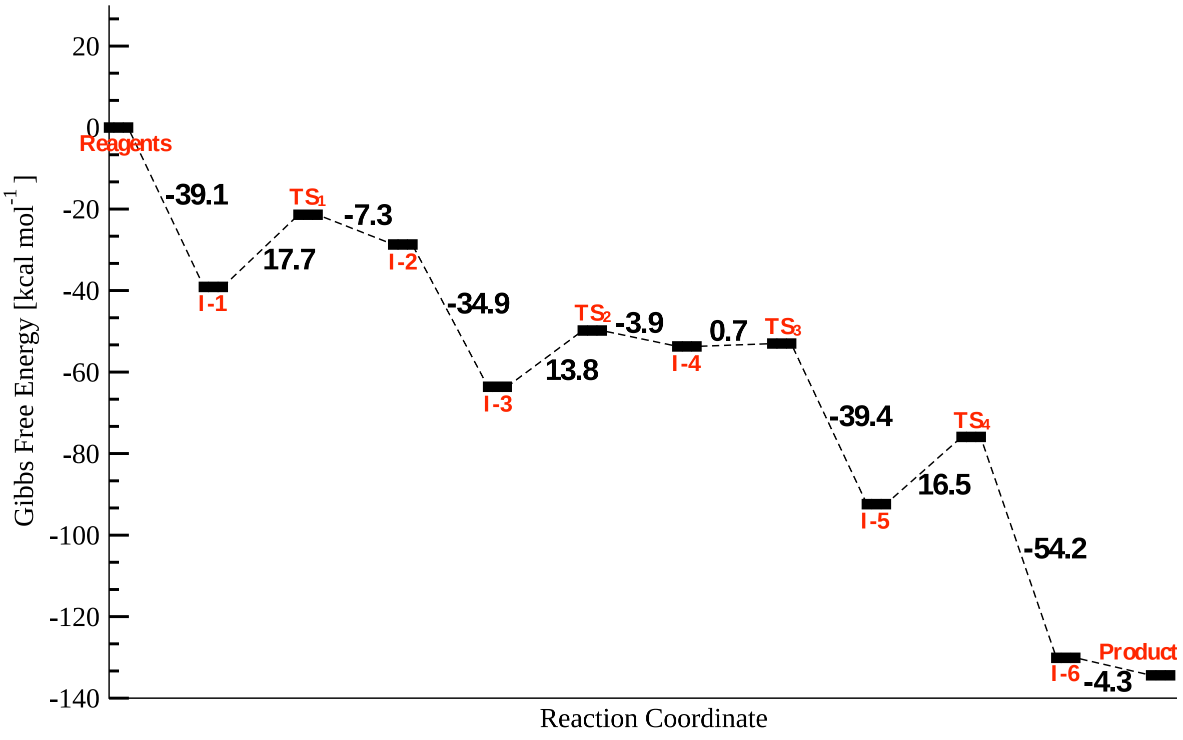

Figure S3. Energy profile for the chlorination of 2-naphthol in the presence of PIDA and $AlCl_3$ (1:2) via the formation of $PhICl_2$. Values represent the Gibbs free energy (in kcal/mol).

Table S3. Structures of intermediates and transition states with their corresponding Gibbs free energies (in kcal/mol) for the chlorination of 2-naphthol in the presence of PIDA - $AlCl_3$ (1:2) via the formation of $PhICl_2$.

Step	Intermediate (I) / Transition State (TS)	ΔG [kcal/mol]
Reagents	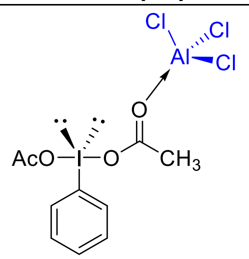	0.0

**I-1**	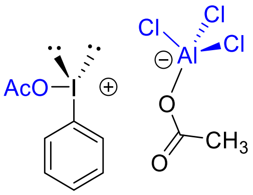	-39.1
**TS$_1$**	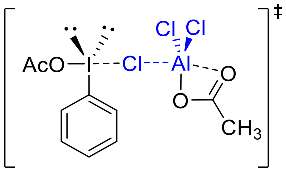	-21.4
**I-2**	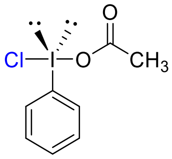	-28.7
**I-3**	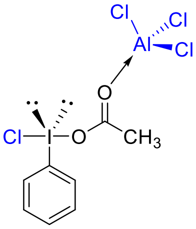	-63.6
**TS$_2$**	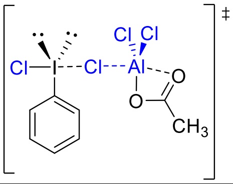	-36.5
**I-4**	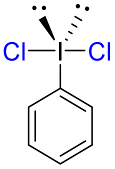	-57.1
**TS$_3$**	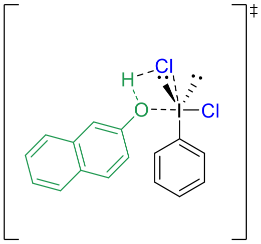	-35.6
**I-5**	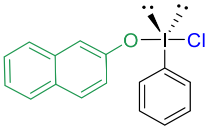	-85.4
**TS$_4$**	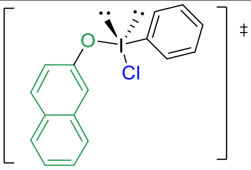	-68.9

**I-6**	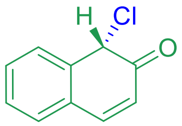	-123.1
Product	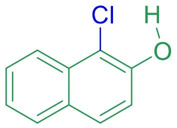	-127.3

5. Alternative mechanisms explored for the bromination of 2-naphtol

a. The PIFA-$AlBr_3$ (1:2) system

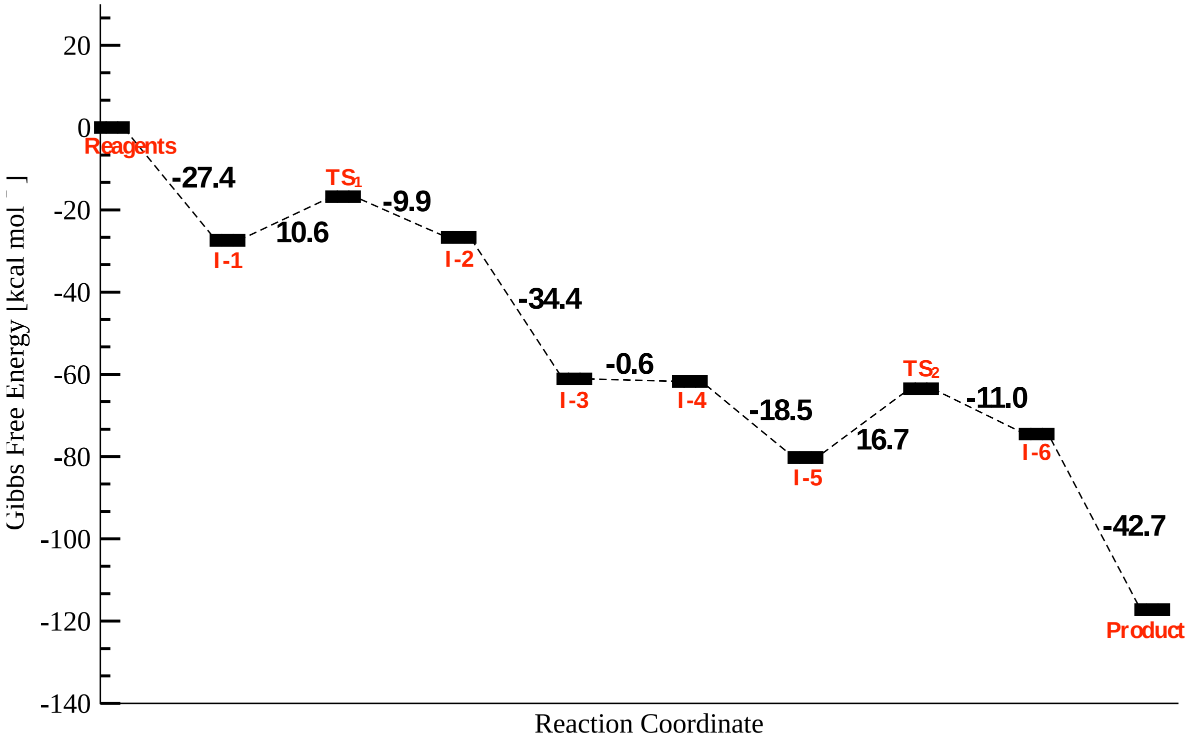

Figure S4. Energy profile for the bromination of 2-naphthol in the presence of PIFA and $AlBr_3$ (1:2). Values represent the Gibbs free energy (in kcal/mol).

Table S4. Structures of intermediates and transition states with their corresponding Gibbs free energies (in kcal/mol) for the bromination of 2-naphthol in the presence of PIFA - $AlBr_3$ (1:2).

Step	Intermediate (I) / Transition State (TS)	ΔG [kcal/mol]
Reagents	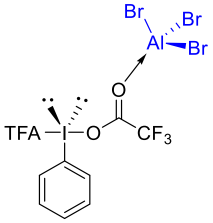	0.0
**I-1**	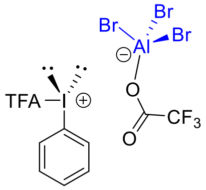	-27.4

**TS₁**	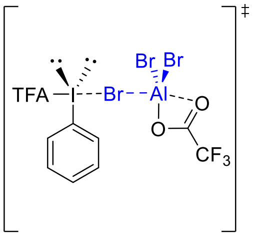	-16.8
TFOAc-AlBr2	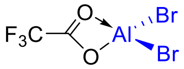	
**I-2**	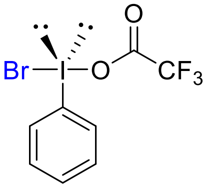	-26.7
**I-3**	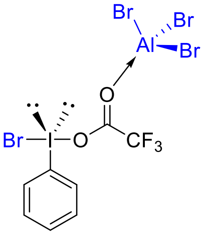	-61.1
**I-4**	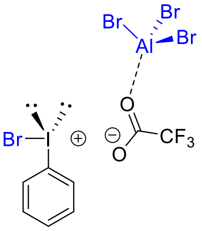	-61.7
**I-5**	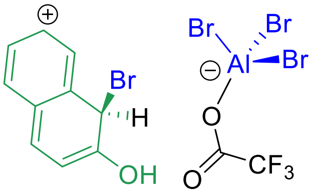	-80.2
**TS₂**	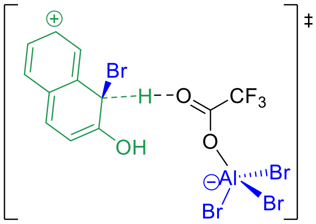	-63.5
**I-6**	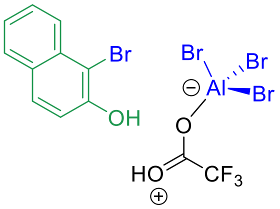	-74.5

Product	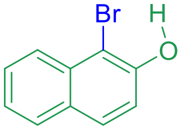	1-Br-2-NpOH

5. Alternative mechanisms explored for the bromination of 2-naphtol

b. The PIFA-$AlBr_3$ (1:2) system via the formation of $PhIBr_2$

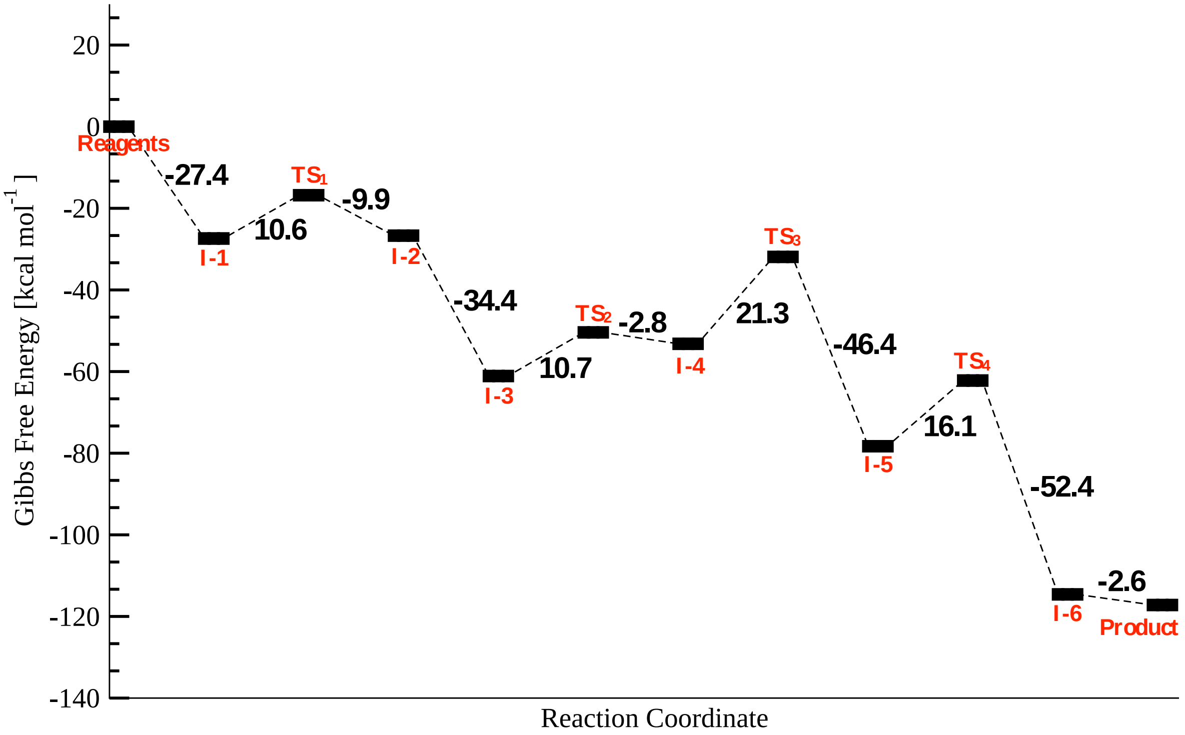

Figure S5. Energy profile for the bromination of 2-naphthol in the presence of PIFA and $AlBr_3$ (1:2). Values represent the Gibbs free energy (in kcal/mol) via the formation of $PhIBr_2$.

Table S5. Structures of intermediates and transition states with their corresponding Gibbs free energies (in kcal/mol) for the chlorination of 2-naphthol in the presence of PIFA - $AlBr_3$ (1:2), via the formation of $PhIBr_2$.

Step	Intermediate (I) / Transition State (TS)	ΔG [kcal/mol]
Reagents	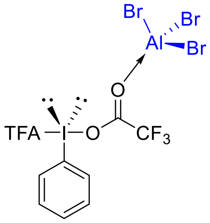	0.0
**I-1**	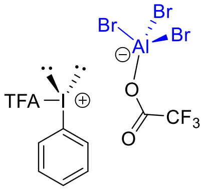	-27.4

**TS_1**	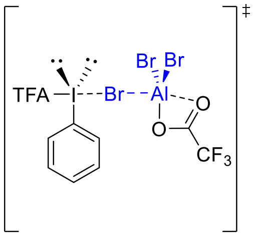	-16.8
**I-2**	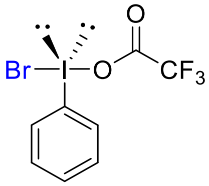	-26.7
**I-3**	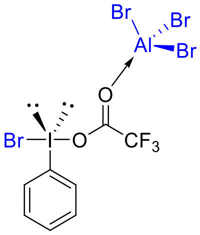	-61.1
**TS_2**	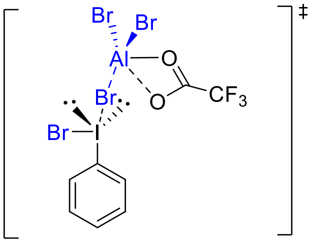	-50.4
**I-4**	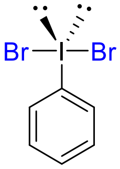	-53.2
**TS_3**	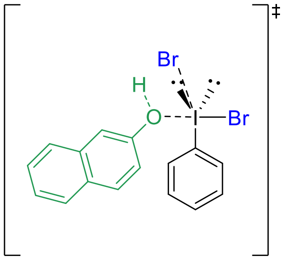	-31.9
**I-5**	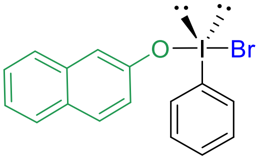	-78.3

**TS$_4$**	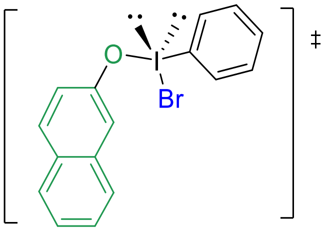	-62.2
**I-6**	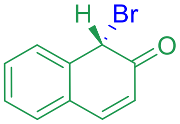	-114.6
Product	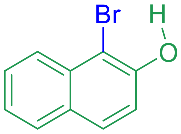	-117.2

5. Alternative mechanisms explored for the bromination of 2-naphtol

c. The PIDA-$AlBr_3$ (1:2) system via the formation of $PhIBr_2$

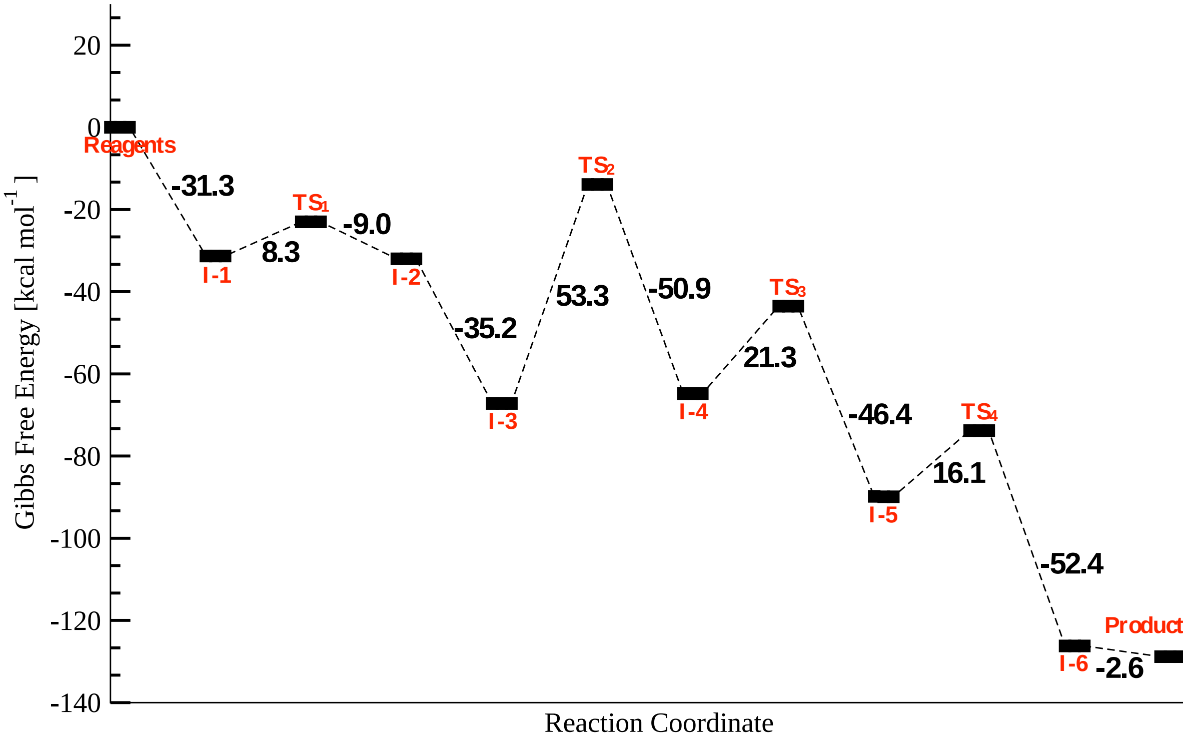

Figure S6. Energy profile for the bromination of 2-naphthol in the presence of PIDA and $AlBr_3$ (1:2), via the formation of $PhIBr_2$, values represent the Gibbs free energy (in kcal/mol)

Table S6. Structures of intermediates and transition states with their corresponding Gibbs free energies (in kcal/mol) for the bromination of 2-naphthol in the presence of PIDA - $AlBr_3$ (1:2), via the formation of $PhIBr_2$.

Step	Intermediate (I) / Transition State (TS)	ΔG [kcal/mol]
Reagents	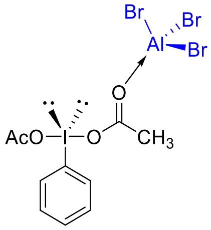	0.0
**I-1**	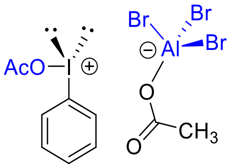	-31.3
**TS₁**	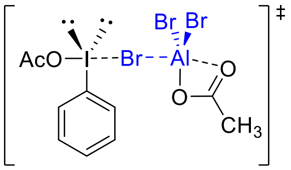	-23.0

**I-2**	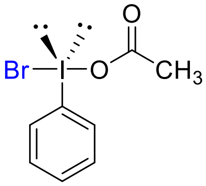	-32.0
**I-3**	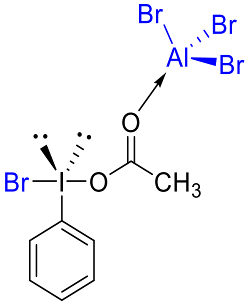	-67.2
**TS_2**	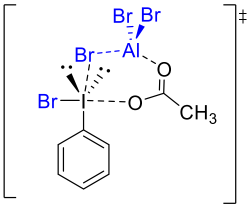	-13.9
**I-4**	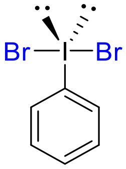	-64.8
**TS_3**	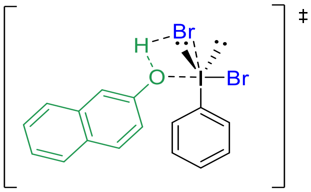	-3.5
**I-5**	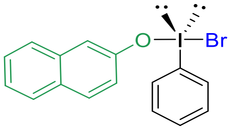	-89.9
**TS_4**	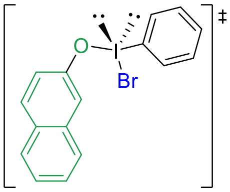	-73.8
**I-6**	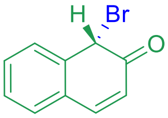	-126.2
Product	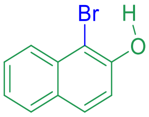	1-Br-2-NpOH
